# The Effects of Green and Urban Walking in Different Time Frames on Physio-Psychological Responses of Middle-Aged and Older People in Chengdu, China

**DOI:** 10.3390/ijerph18010090

**Published:** 2020-12-24

**Authors:** Hansen Li, Haowei Liu, Zongqian Yang, Shilin Bi, Yang Cao, Guodong Zhang

**Affiliations:** 1 Institute of Sports Science, Key Lab of Physical Fitness Evaluation and Motor Function Monitoring of General Administration of Sports of China, College of Physical Education, Southwest University, Chongqing 400715, China; lhs510416413@email.swu.edu.cn (H.L.); haoweiliu@email.swu.edu.cn (H.L.); Yzq000133@email.swu.edu.cn (Z.Y.); 2 National Institute of Education, Nanyang Technological University, Singapore 637616, Singapore; NIE20.BS@E.NTU.EDU.SG; 3 Clinical Epidemiology and Biostatistics, School of Medical Sciences, Örebro University, 70182 Örebro, Sweden; 4 Unit of Integrative Epidemiology, Institute of Environmental Medicine, Karolinska Institutet, 17177 Stockholm, Sweden

**Keywords:** walking, green space, urban, nighttime, blood pressure, physiological, psychological

## Abstract

Nighttime walking is becoming a popular exercise for many middle-aged and older people in Asian countries. However, the health benefits of nighttime walking in urban areas and green spaces are still unclear. This study evaluated the physiological and psychological responses of 48 middle-aged and older people who walked 1.6 km through a green space and an urban area during daytime and nighttime. The Positive and Negative Affect Schedule (PANAS), Profile of Mood States (POMS), Perceived Restorativeness Scale (PRS), and Restorative Outcome Scale (ROS) were employed to measure the psychological responses, and pulse rate and blood pressure (SBP, DBP and MAP) were measured to evaluate the physiological responses. The results showed that the daytime green walking induced psychological improvements and lowered blood pressure (*p* < 0.05), while the daytime urban walking resulted in slight deterioration of all the measured parameters (*p* > 0.05). On the other hand, the nighttime green walking induced lowered blood pressure (*p* < 0.05), whilst the nighttime urban walking resulted in psychological improvements and lowered blood pressure (*p* < 0.05), and no significant difference was found in any measured parameter between the two nighttime walking groups. In conclusion, urban areas are noisy and irritating in the daytime, and not suitable for walking, but may become pleasurable and attractive at night. The psychological benefits of green walking may decrease at night, and nighttime walking in either an urban area or a green space may achieve similar health benefits. Therefore, we recommend that urban citizens start nighttime walking in a green space or an urban area to keep fit when the air is less polluted.

## 1. Introduction

As populations become increasingly urbanized, urban living is associated with a series of stressors, including noise, air pollution, and crowding [1,2,3], which have resulted in a range of negative effects on public health, such as the increased risk of cardiovascular diseases and mental problems [4,5]. In response, various attempts have been made to relieve stress and maintain health, and green walking is one of the important solutions, which refers to walking in forests or other natural environments with plants [6]. As both natural settings and physical activities have been considered to benefit physical and mental health [7,8,9,10], the green walking has thus been expected to bring additional health benefits [11].

Numerous studies have found that walking in forests or urban parks could lower blood pressure, increase parasympathetic activities, and improve emotional outcomes [12,13,14,15]. On the contrary, walking in urban areas seems to be less attractive and lack health benefits [16,17,18]. However, there was evidence that the physical and psychological responses could be related to the characteristics of environments, and some urban scenes may also exert positive effects on physical exercise [19], which has implied the possibility of urban surroundings in improving physio-psychological health. For instance, potential health benefits derive from walking through a clean and orderly street, or exercise in a city with less air pollution. Although the importance of nature is irrefutable, the worldwide urbanization makes it harder for people to reach forests or other completely natural environments, and there is thus a rising demand to explore places with restorativeness and positive effects among cities [20].

In recent years, more and more urban citizens choose walking as their daily exercise, especially those middle-aged and older people, who prefer moderate exercises without technical difficulty [21,22]. For most citizens who are employed, nighttime walking is one of their best options. For one, a few hours after dinner are their only time available to exercise. For another, the nighttime exercise may be more effective in lowering blood pressure [23], and may also help to moderate nocturnal blood pressure in active people [24]. Besides, mild exercise, like walking, is unlikely to be a threat to sleep quality [25]. In fact, nighttime exercises are popular in many Asian countries due to the local cultures, and a vast number of people have already got involved in the nighttime walking [26,27], which may be considered as an easy and safe exercise owing to the bright urban illumination, the fine walking facilities, and the good public order. Many middle-aged and older people in China usually go on nighttime walking together, which not only meets their needs of keeping fit, but also provides opportunities for social communication, thus make them adhere to long-term physical exercise. In terms of nighttime walking environment, the urban citizens today can easily access to various green spaces owing to the advanced urban greening [28,29]. Nevertheless, there are quite a few people who tend to carry out nighttime walking in urban areas instead of green spaces, and the reasons were unknown. Whereas the daytime green walking and urban walking have been well studied, less attention has been paid to the cases in the nighttime, and the characteristics of the two nighttime environments remain to be further investigated.

Theoretically, visual experience plays an important role in green walking [30], and greenness of plants can help to achieve positive changes in physical and mental indices [31]. Therefore, improper lighting at night may interfere with the cognition of greenness and thus reduce the effects from natural elements. Moreover, plants are the key for carbon sequestration and oxygen generation [32], which may exert positive impacts on the respiratory and related nervous system. However, the photosynthesis is becoming inactive at night, and plants’ respiration may even lead to higher carbon dioxide concentration, which may also reduce the health benefits brought by the green walking. On the other hand, urban areas have better illumination, the artificial lights in cities may improve the scenic experience and sense of safety [33], and further add joy to nighttime recreations. Besides, the traffic emission, one of the main street air pollutants that prevent people from urban activities, may reduce at night, and so do the noise and crowding, which may make nighttime urban walking more comfortable and enjoyable. Thereby, our experimental study was aimed to assess the effects of green walking and urban walking in daytime and nighttime, and further reveal the differences between the nighttime walking in green space and urban area. The specific hypotheses were:

(1) The daytime urban walking has negative effects while the nighttime urban walking has positive effects on the investigated psychological and physiological responses;
(2) The daytime green walking has positive effects while the nighttime green walking has negative effects;
(3) Compared to the nighttime green walking, the urban walking has greater positive effects and is more attractive to urban citizens.

## 2. Materials and Methods

### 2.1. Participants

The inclusion criteria for recruiting the middle-aged and older people who were eligible for the study are:

(1) Aged between 40 and 75 years.
(2) Absence of serious cardiovascular disease.
(3) Absence of chronic symptoms causing walking problems.
(4) Absence of cognitive impairment and mental disorders.

As a result, 48 people (40–71 years old) were recruited from a community in Chengdu, Sichuan Province, China. Participants were informed of experimental procedures and the possible risks and benefits of their participation. All participants were randomly divided into the following four groups by computer (Table 1). There was no statistically significant difference in age or BMI index among the different groups (analyzed using Kruska–Wallis test).

(1) Daytime green walking (**DG**).
(2) Nighttime green walking (**NG**).
(3) Daytime urban walking (**DU**).
(4) Nighttime urban walking (**NU**).

As analyzed in a previous study, a sample size between 9 and 19 participants is enough to achieve acceptable statistical power for the predefined hypotheses [12], therefore our sample size is appropriate. All experimental procedures undertaken in the present study were under regulations of and approved by the Ethics Committee of the Physical Education College of Southwest University, China.

### 2.2. Study Sites

**Figure 1:**
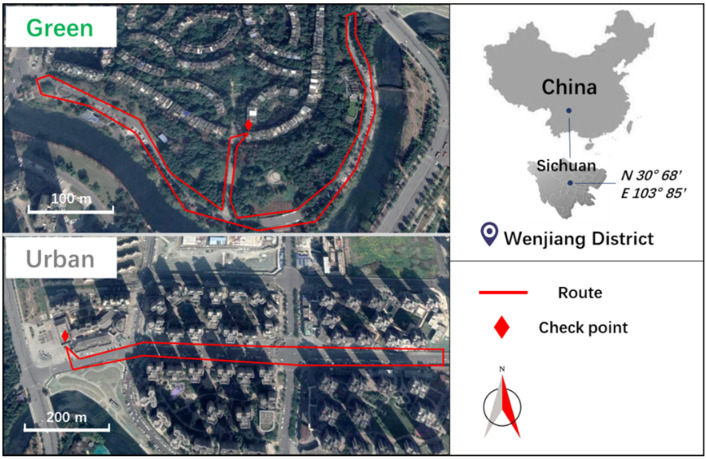
Location of the study area and the two walking routes in the green space and urban area.

After a field investigation, a green space and an urban area in Wenjing District, Chengdu, were selected for walking (Figure 1). Both sites are flat without slope, and less than half a kilometer from the participants’ community. The green space is located in the middle of two residential zones, decorated with trees, shrubs, and grass, and close to a river. The urban area contains a street among commercial and residential zones, decorated with a few trees, and there are wide pedestrian areas on both sides of the street.

**Figure 2:**
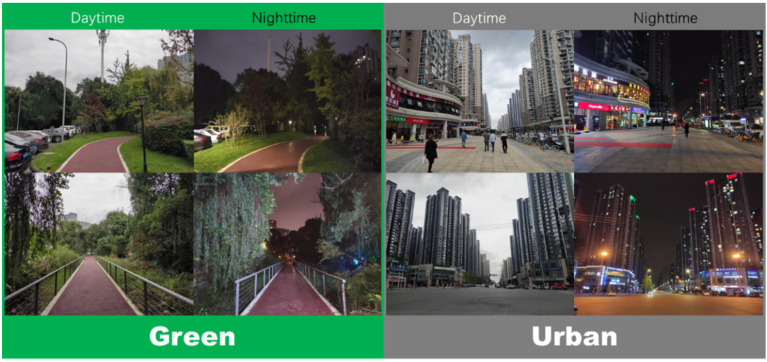
The main views of the two walking routes in daytime and nighttime.

In order to ensure the safety of the participants, two circular routes (1.6 km each) were set up in flat and illuminated walkways among the green space and urban area, respectively, which need approximately 20 min to finish (Figure 1). Besides, three staff were arranged to stay in the routes to monitor the whole walking program, and a community doctor was also hired to stay at the check point to provide medical attention. The main views of the two routes in daytime and nighttime are demonstrated in Figure 2. An indoor check point was established in each route for measurements of pre-tests and post-tests.

### 2.3. Process and Contents of Walking Experiment

The experiment was carried out in October. As the experiment sites were in the participants’ neighborhoods, the participants were asked to reach the check points on their own before the beginning of the experiment. Thereafter, all the participants were seated in the waiting rooms for a half-hour rest. During the rest, the participants were provided with route maps and informed of walking and measurement details by the research staff. The experiment was introduced as self-paced walking, and the participants were asked to walk alone at their easy and comfortable speeds. Psychological questionnaires, measurements of blood pressure, and measurement of pulse rate were completed before and after the walking (Table 2).

The data of illumination, temperature, and noise were collected every 300 m of the routes using a light detector (Suwei-SW6013), an ambient thermometer (SmartSensor-AS817), and a cell-phone noise detection software, respectively. In addition, the real-time air quality index was also obtained from the website of the Chengdu Meteorological Bureau. The relevant data are shown in Table 3.

After the walk, an interview was conducted to evaluate the preference of participants. After confirming that all the participants were familiar with the nighttime and daytime images of the study area, the participants were required to make two choices, one was to choose between the daytime urban area and daytime green space, another was to choose between the nighttime urban area and nighttime green space, and the reasons for their like or dislike were also asked and recorded.

### 2.4. Measurement of the Outcomes

#### 2.4.1. Physiological Parameters

Systolic blood pressure (SBP), diastolic blood pressure (DBP), and pulse rate were measured using portable electronic sphygmomanometers (OMRON HEM-7211) at the check points before and after the walk. Measurements were performed in a relaxed sitting position and the instrument was placed at heart height. The mean arterial pressure (MAP) was calculated as ((DBP × 2) + SBP)/3.

#### 2.4.2. Psychological Parameters

Four psychological scales were employed to measure the emotional outcomes and restorative effects.

The Positive and Negative Affect Schedule (PANAS), which consists of 20 items and is evaluated by a Likert five-point scale, is widely used to evaluate changes in positive and negative emotions [34,35]. The reliability and validity of PANAS were tested in previous studies [36,37]. A validated Chinese version of PANAS scale was employed in this study [38].

A simplified Chinese version of Mood States questionnaire (POMS), which contains 40 items and is evaluated by a four-point Likert scale, was employed to measure personal moods. The Chinese version has been proved to be suitable for the Chinese population, and has been widely used [39,40]. Results of the POMS scale can help to explain six subscales of mood: Tension or anxiety (T), anger or hostility (A), fatigue (F), depression or dejection (D), vigor (V), and confusion or bewilderment (C). Besides, the Chinese POMS contains 5 items (items 7, 14, 27, 34, and 40) that are related to self-esteem. Meanwhile, the total mood disturbance (TMD) can be evaluated from the subscales (TMD = the sum of negative emotions − the sum of positive emotions + 100).

Restorativeness is defined as the potential of certain surroundings for restoring certain cognitive capacities related to human information [41]. Two relevant scales were used in the present study to observe the restorative effects of environments.

A shortened version of the Perceived Restorativeness Scale (PRS) was used to measure perceived restorativeness of environment based on the Attention Restoration Theory [42,43,44]. The reliability and validity of the short scale were tested previously [45]. The scale consists of five items with a 10-point Likert scale.

The Restorative Outcome Scale (ROS), which contains six items and is evaluated by a seven-point Likert scale, was used to evaluate properties of the environments that contributed to the restorative outcomes. According to previous research, the scale has been confirmed with high reliability and validity. [46,47].

As there are no Chinese version of the short PRS and ROS, they were translated into Chinese, and checked by back-and-forth translation in our study.

### 2.5. Statistical Methods

All the data were processed via SPSS 25.0 (SPSS Inc., Chicago, IL, USA). Given our sample size, the Shapiro–Wilk test was employed to determine the distribution of all data. Due to some data that were hard to normalize, the nonparametric test was employed for non-normal data. When comparing the difference between pre- and post-test, the paired t-test was employed to compare normal data, and the Wilcoxon signed-rank test was employed to compare non-normal data. When comparing the difference between multiple scenarios, if normal distribution and variance homogeneity were confirmed, then the one-way ANOVA was employed, and followed with post hoc comparisons using the Duncan’s multiple range test. On the other hand, the Kruskal–Wallis test was employed for comparisons of non-normal data between different groups. When determining the effects of time and sites, if the data were normally distributed and consistent with homogeneity of variance, the two-way ANOVA was performed. If there was a significant interaction, the simple effect was tested using the post hoc LSD method. The Fisher’s exact test was performed to check the difference in the proportion of participants with a positive response between different groups, and followed with post hoc multiple tests adjusted with Bonferroni correction. The Chi-squared test was employed to check the difference in participants’ preference between the two walking environments. A *p*-value < 0.05 was considered statistically significant in the present study.

## 3. Results

### 3.1. Validation of the Psychological Measurements

The Cronbach’s α showed good internal consistency (>0.7) for the PANAS and PRS in the all scenarios (Table 4). In the case of the POMS, though good reliabilities were found in nighttime green walking, daytime urban walking, and nighttime urban walking, the coherence in daytime green walking did not meet a satisfactory level (Cronbach’s α = 0.56). Moreover, the reliabilities of ROS in daytime green walking (Cronbach’s α = 0.62) and nighttime green walking (Cronbach’s α = 0.69) were barely within the acceptable level (Cronbach’s α between 0.6 and 0.7).

### 3.2. Pyschological Outcomes of Walking in Different Scenarios

**Figure 3:**
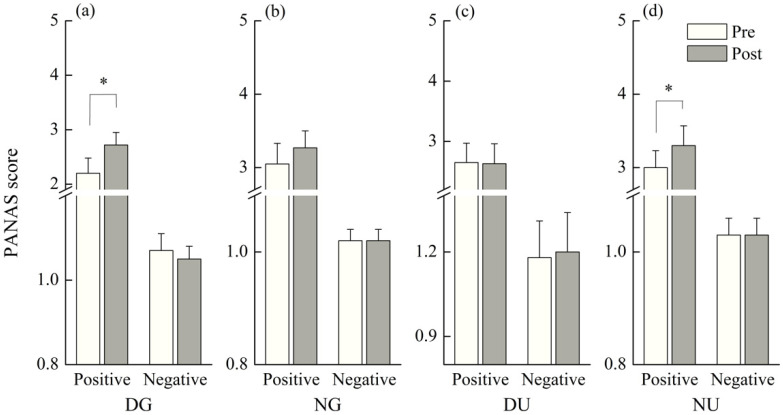
Outcomes of Positive and Negative Affect Schedule (PANAS) in: (**a**) daytime green walking; (**b**) nighttime green walking; (**c**) daytime urban walking; and (**d**) nighttime urban walking. The error bar represents standard error, and * represents *p* < 0.05. Analyzed using Wilcoxon signed-rank test (*N* = 12).

The median score of PANAS-positive significantly increased after daytime green walking (*p* = 0.03) and nighttime urban walking (*p* = 0.046), and the value after nighttime green walking also increased but not statistically significantly (*p* > 0.05) (Figure 3). However, no statistically significant change was found in PANAS-negative score of any scenario (Figure 3).

**Figure 4:**
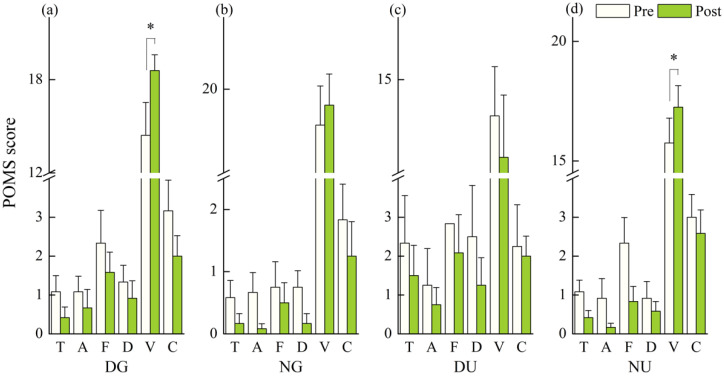
Outcomes of Profile of Mood States (POMS) subscales in: (**a**) daytime green walking; (**b**) nighttime green walking; (**c**) daytime urban walking; and (**d**) nighttime urban walking. T: Tension or anxiety; A: Anger or hostility; F: Fatigue; D: Depression or dejection; V: Vigor; C: Confusion or bewilderment. The error bar represents standard error, and * represents *p* < 0.05. Analyzed using Wilcoxon signed-rank test (*N* = 12).

In terms of the POMS subscales, the median scores of tension-anxiety, anger-hostility, fatigue, depression-dejection, and confusion-bewilderment slightly decreased after walking in all scenarios without statistical significance (*p* > 0.05). The median score of vigor significantly increased after daytime green walking (*p* = 0.034) and nighttime urban walking (*p* = 0.024) (Figure 4a,d).

**Figure 5:**
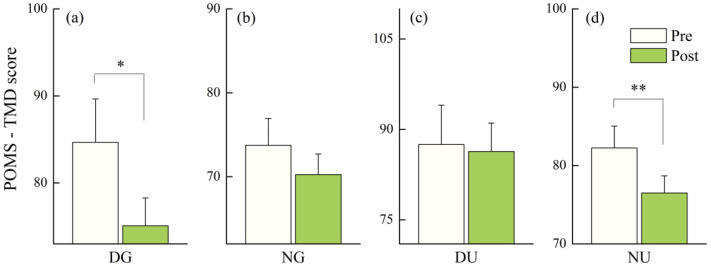
Outcomes of POMS-total mood disturbance (TMD) in: (**a**) daytime green walking; (**b**) nighttime green walking; (**c**) daytime urban walking; and (**d**) nighttime urban walking. The error bar represents standard error, and * represents *p* < 0.05, ** represent *p* < 0.01. Analyzed using Wilcoxon signed-rank test (*N* = 12).

The median score of TMD decreased in all scenarios, but only the decreases after daytime green walking (*p* = 0.018) and nighttime urban walking (*p* = 0.003) were statistically significant (Figure 5a,d).

**Figure 6:**
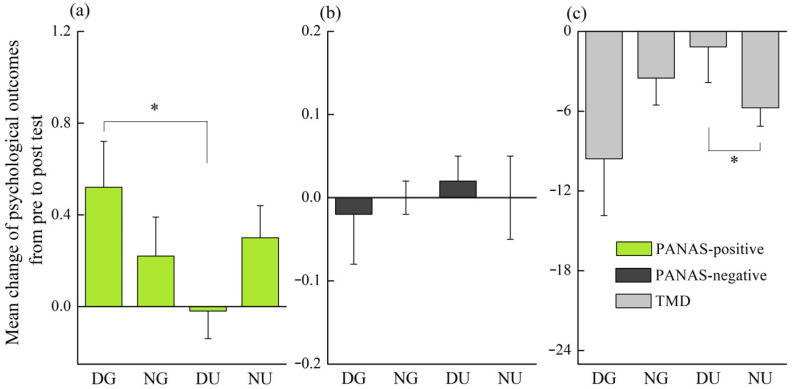
The psychological changes of walking in different scenarios. The (**a**), (**b**), and (**c**) represent changes in scores of PANAS-positive, PANAS-negative, and POMS-TMD respectively. The DG, NG, DU, and NU represent daytime green walking, nighttime green walking, daytime urban walking, and nighttime urban walking respectively. The error bar represents standard error, and * represents *p* < 0.05. Analyzed using the Kruskal–Wallis test (*N* = 12).

Due to the non-normal psychological data, the mean changes of measured parameters were calculated to reveal the differences of walking in different scenarios. The psychological changes demonstrated that the increase in score of PANAS-positive in daytime green walking was significantly greater than that in daytime urban walking (*p* = 0.032) (Figure 6a). The decrease in score of POMS-TMD in nighttime urban walking was significantly greater than that in daytime urban walking (*p* = 0.02) (Figure 6c). No statistically significant difference was found in the psychological changes between nighttime green walking and nighttime urban walking.

The proportions of participants who exhibited positive responses were also calculated to reveal the difference among different walking scenarios. However, no statistically significant difference was found among the scenarios (Table 5).

### 3.3. Restorativeness of Different Walking Environments

**Figure 7:**
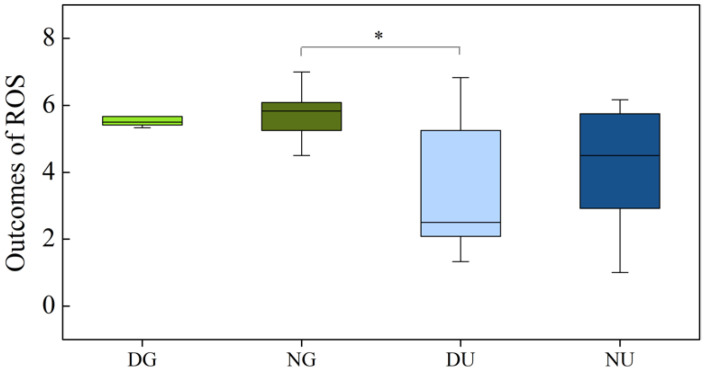
Restorative outcomes of Restorative Outcome Scale (ROS) in different scenarios. The DG, NG, DU, and NU represent daytime green walking, nighttime green walking, daytime urban walking, and nighttime urban walking, respectively. The * represents significant differences (*p* < 0.05). Analyzed using the Kruskal–Wallis test (*N* = 12).

The restorativeness of the environments was assessed using the PRS and ROS scales after the walks. Similar results were observed in the two scales, and a significant correlation was found between the two measurements (Pearson’s r = 0.834, *p* < 0.001). Due to the non-normal distribution of ROS outcomes, the Kruskal–Wallis test was performed and revealed a statistically significant difference between nighttime green walking and daytime urban walking (*p* = 0.022) (Figure 7).

In the case of PRS, the two-way ANOVA revealed that there was a statistically significant difference between sites, but no significant difference between time frames (Table 6). A significant interaction between time frames and sites was also observed (Table 6).

**Figure 8:**
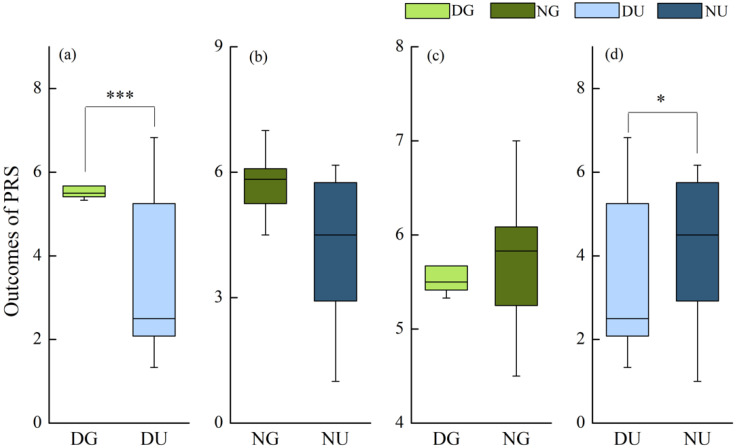
Restorative outcomes of Perceived Restorativeness Scale (PRS) in: (**a**) daytime walking; (**b**) nighttime walking; (**c**) green walking; and (**d**) urban walking. The * represents *p* < 0.05, and *** represents *p* < 0.001. Compared using Post Hoc Multiple comparisons (LSD method) (*N* = 12).

The analysis of simple effects revealed that during the daytime, the score of PRS in green walking was significantly higher than that in urban walking (*p* < 0.001) (Figure 8a), but no statistically significant difference was found between them during nighttime (Figure 8b). In the urban walking, the score of PRS in nighttime walking was significantly higher than that in daytime walking (*p* = 0.037) (Figure 8d), but no significant difference was found between the time frames in green walking (Figure 8c).

### 3.4. Physiological Outcomes of Walking in Different Scenarios

**Figure 9:**
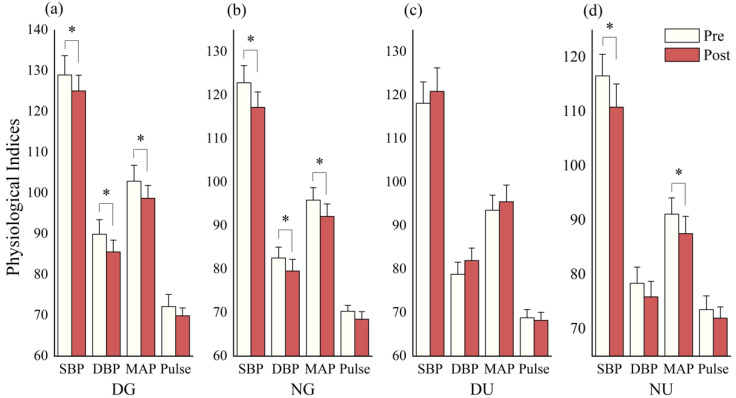
Outcomes of physiological measurements in: (**a**) daytime green walking; (**b**) nighttime green walking; (**c**) daytime urban walking; and (**d**) nighttime urban walking. The error bar represents standard error, and * represent *p* < 0.05. Analyzed using Paired t-test (*N* = 12).

The SBP significantly decreased after daytime green walking (*p* = 0.038), nighttime green walking (*p* = 0.005), and nighttime urban walking (*p* = 0.006). The DBP significantly decreased after daytime green walking (*p* = 0.006) and nighttime green walking (*p* = 0.009). The MAP significantly decreased after daytime green walking (*p* = 0.008), nighttime green walking (*p* = 0.005), and nighttime urban walking (*p* = 0.045). However, no statistically significant change difference was found in the pulse rate in any scenario (Figure 9).

**Figure 10:**
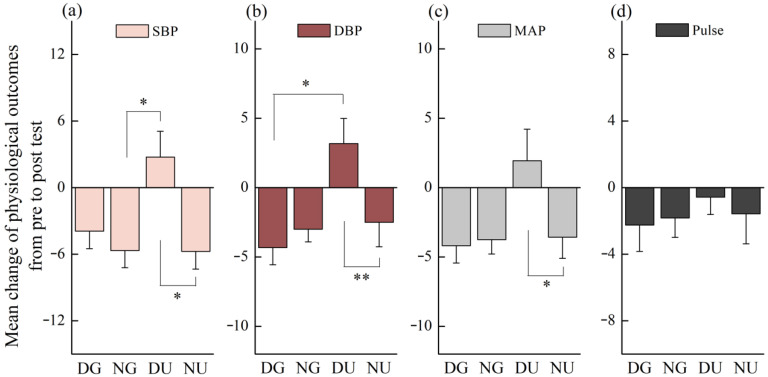
The physiological changes of walking in different scenarios. The (**a**), (**b**), (**c**), and (**d**) represent changes in SBP, DBP, MAP, and pulse rate respectively. The DG, NG, DU, and NU represent daytime green walking, nighttime green walking, daytime urban walking, and nighttime urban walking, respectively. The error bar represents standard error, * represents *p* < 0.05, and ** represents *p* < 0.01. Analyzed using the Kruskal–Wallis test (*N* = 12).

Due to the diurnal variation of physiological parameters, the mean changes of physiological outcomes were calculated to reveal the differences in walking in different scenarios. The physiological changes demonstrated that the decreases of SBP in both nighttime green walking (*p* = 0.04) and nighttime urban walking (*p* = 0.048) were significantly greater than that in daytime urban walking (Figure 10a). The decreases of DBP in both daytime green walking (*p* = 0.06) and nighttime green walking (*p* = 0.03) were significantly greater than that in daytime urban walking (Figure 10b). The decrease of MAP in nighttime urban walking was significantly greater than that in daytime urban walking (*p* = 0.033) (Figure 10c). No statistically significant difference was found in the physiological changes between nighttime green walking and nighttime urban walking.

The Fisher’s Exact Test revealed statistically significant differences in proportions of participants with positive responses in SBP (*p* = 0.002), DBP (*p* = 0.005), and MAP (*p* = 0.004) among different scenarios (Table 7).

### 3.5. Walking Environment Preference of Participants

**Figure 11:**
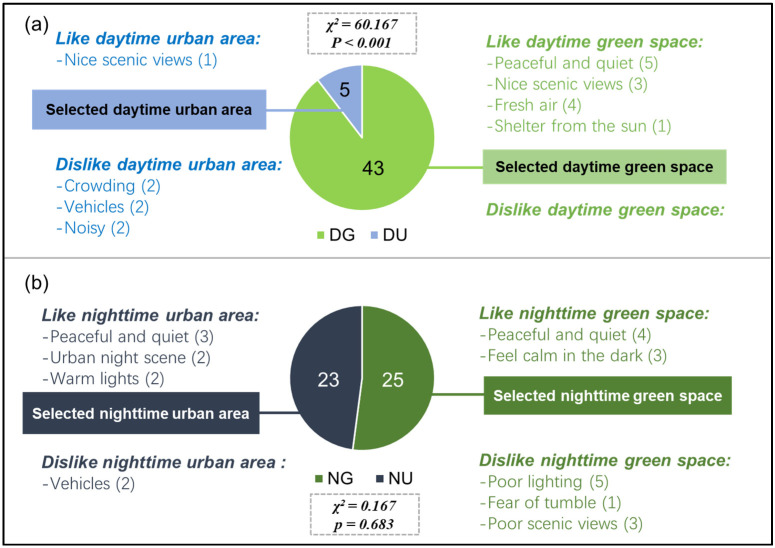
Preferences of participants towards different walking environments. The (**a**) and (**b**) represent daytime walking environments and nighttime walking environments respectively. The DG, NG, DU, and NU represent daytime green space, nighttime green space, daytime urban area, and nighttime urban area, respectively. Analyzed using Chi square test (*N* = 48).

In terms of daytime walking environments, 93.7% of the participants preferred the daytime green space (Figure 11a). However, in terms of nighttime walking environments, only 52.1% of participants chose the nighttime green space (Figure 11b). In the interview, only some participants could give explicit reasons for their options, and these reasons could be classified into several points (Figure 11).

## 4. Discussion

The present study aimed to check how green walking and urban walking in different time frames affect the physiological and psychological characteristics of the participants. The main findings indicated that both the daytime green walking and nighttime urban walking induced significant positive changes in blood pressure and psychological outcomes, and the nighttime green walking also induced lowered blood pressure. The daytime urban walking was inferior to the other three scenarios in psychological or physiological improvements, but no significant difference was found among the daytime green walking, nighttime green walking, and nighttime urban walking.

### 4.1. Urban Walking

In the present experimental study, blood pressure (SBP, DBP, and MAP), and some psychological parameters (PANAS-positive and POMS-Vigor) demonstrated reduction after the daytime urban walking. In contrast, the nighttime urban walking was associated with significant improvements in moods (PANAS-positive, POMS-vigor, and POMS-TMD) and blood pressure (SBP and MAP). In addition, the nighttime PRS score of the urban area was significantly higher than the daytime counterpart. These findings supported our first hypothesis that the daytime urban walking has negative effects while the nighttime urban walking has positive effects. Since the two walking options were carried out in the same area, these differences might be due to the change of the properties of the urban environment at night.

Compared with previous works, our results of daytime urban walking agreed with the finding of Ji, et al. [14], Hassan, et al. [48], and Song, et al. [49], who have also reported negative effects of urban environments on blood pressure as well as some psychological outcomes. However, there is a difference between the participants: Our study only included middle-aged and older people, while the above studies only included young participants, which indicates that the daytime urban environment may have negative impacts on people of different ages. Besides, these negative impacts, according to Ji, et al. [14], and Hassan, et al. [48], were caused by increased stress levels in urban surroundings, and the stress responses were detected in the test of amylase and brain wave. Theoretically, physical activity can benefit physiological and psychological health [10,50,51], and walking, the basic exercise that we were concerned with, has been proved to lower blood pressure and improve moods [50,51]. However, the health benefits of physical activity may be counteracted by urban stressors such as noise, crowding, and pollution [52,53]. For example, the autonomic nervous system, an important regulator for peripheral resistance and cardiac outputs [54], can be inhibited by certain traffic noise, and thus increase blood pressure [55,56,57]. In the following interview of the present study, these urban conditions were also considered as unpleasant factors by some participants (Figure 11). Therefore, the poorer effects observed in the daytime urban walking suggest that the daytime urban environment exerts a dominant influence, which might attenuate or even mask the benefits of walking.

In terms of the nighttime urban walking, responses were found more positive. In a relevant study, Fullick, et al. [24] found that a night exercise carried out indoors resulted in postexercise hypotension, which was similar to our results, indicating that the effects of physical activity remained in the outdoor urban environment. Based on the field investigation, a significant decrease in noise was observed in the nighttime urban area (Table 3). Meanwhile, according to our observation during the experiment, the pedestrians were obviously fewer at night, which could also reduce feelings of being exposed and make participants feel safe and comfortable [58]. Besides, some participants in our study also mentioned that the urban area was quiet and peaceful at night (Figure 11). These changes might partly explain the better responses at night. Moreover, another important reason might be the enhanced safety and visuality owing to artificial lighting [33]. Despite current concerns over light pollution, artificial lights do bring convenience to the urban citizen’s nighttime activities. Cajochen, et al. [59] has reported that exposure to commercial light at 6500K resulted in enhanced subjective alertness, well-being, and visual comfort. Another study further emphasized the psychological experiences affected by light properties, and reported that the exposure to an artificial morning dawn simulation light improved participants’ well-being, mood, and cognitive performance [60]. Our data showed that the urban street had the best illumination in all scenarios (Table 3), and the lights from streetlights and shops were generally warm, which made some participants feel warm and comfortable (Figure 11). Overall, our findings confirm the negative impacts of the daytime urban area, but indicate that nighttime urban walking may have positive effects. Given that many cities have pedestrian streets or areas that are open and free of vehicles, these places at night, especially the quiet ones, are probably suitable places for nighttime walking after work.

### 4.2. Green Walking

In the present study, the daytime green walking has resulted in significant improvements in psychological (PANAS-positive, POMS-vigor, and POMS-TMD) and physiological (SBP, DBP, and MAP) parameters. By comparison, though the psychological improvements were not statistically significant at night, similar positive trends were found, and decreases in blood pressure (SBP, DBP, and MAP) were statistically significant. In terms of the PRS and ROS, both scenarios received similar good scores. These results do not support our second hypothesis that the daytime green walking has positive while the nighttime green walking has negative effects.

In the published literature, the benefits of green walking in lowering blood pressure and improving psychological outcomes have been recorded [12,13,61]. Our findings of daytime green walking have reconfirmed the effects of lowering blood pressure and improving moods. However, there is still a notable difference, the green environments in previous studies were forests or urban parks, in contrast to the urban greenway in our study. Unlike forests or parks with relatively independent large areas, urban green spaces are usually distributed in cities, close to the building area, have smaller green coverage, and may be parked with cars like the present study area (Figure 2). This difference suggests that small-scale or incomplete natural green environments in cities may also exert positive effects on walking exercise. The finding is similar to that by Janeczko, et al. [62] who also concluded that walking in an urban environment with greenery and walking in a forest environment induced similar effects on physiological and psychological relaxation. Taken together, these results further confirm the positive roles of urban green space in stress relief and health improvement.

In terms of the nighttime green walking, the reasons for the non-significant psychological improvements and the decreased blood pressure were still unclear. As speculated previously, visual experience might play an important role in green walking [30]. A simulation by Briki and Majed [31] has confirmed the calming and relaxing effect of the color green on the human organism, which further implied the importance of visual interacting with plants during green walking. As the night went on, though considerable lights were placed in the green space, the illumination situation was still the worst due to the shading of tree crowns (Table 3). Theoretically, the color vision and foveal acuity seriously reduced in nighttime due to characteristics of human rod-mediated vision. Reeves, et al. [63] found that human night vision is very slow to adapt to changes in light levels. Therefore, in addition to insufficient illumination, uneven illumination caused by plant shading may also interfere with visual interacting when walking through the green space. In a similar study, Horiuchi et al. (2014) found that staying in a forest without visual interacting had led to lowered blood pressure, but the outcomes of POMS were not affected. Our findings were very similar to those recorded by Horiuchi et al. (2014), which indicate that visual interacting may play a key role in psychological responses. Considering that some participants disliked the nighttime green space for its poor scenic views (Figure 11), the non-significant psychological responses in nighttime green walking could be partly explained by the insufficient visual interacting with green elements. Moreover, the lowered blood pressure, according to Horiuchi et al. (2014), was possibly induced by other environmental factors such as smell and sound. In the present study, the green space had the lowest noise level (Table 3), which might help participants to stay calm and lower their blood pressure [64]. Besides, some studies have found that smell materials produced by trees may increase parasympathetic activity, thereby lowering blood pressure [65,66]. However, these mechanisms are not confirmed in the present study. Our findings suggest that nighttime green walking may not effectively improve moods, but it is still effective in lowering blood pressure. The green space in cities is still a feasible option for daytime and nighttime walking.

### 4.3. Nighttime Walking

In the present study, nighttime walking in both the green space and urban area induced positive changes, but no statistically significant differences were found in the post-test or the proportion of participants with positive responses. In terms of the PRS and ROS, though the green space scored a little higher than the urban area, the difference is not statistically significant. These results do not support our third hypothesis that urban walking has more positive and is more attractive than green walking at nighttime.

In recent years, green environments have been widely recognized to be more advantageous to human health than urban environments [67,68]. Pratiwi, et al. [12] have found greater improvements in blood pressure (SBP and DBP) and psychological parameters (POMS-TMD and STAI) after walking in an urban park than walking in the city. Likewise, similar results were also reported in other walking studies [49,69]. However, these differences were not seen in the nighttime green walking, which might be explained by the changes in environmental properties as we discussed above. These results indicate the similar benefits of nighttime walking in urban areas and green spaces.

In addition, the results of restorativeness were not in accordance with the findings by Sonntag-Öström, et al. [58], who reported that four types of green environments all scored much higher than a city environment. There was an assumption that urban environments were inherently lacking stress-reducing and mood-enhancing functions [18], and the restorative effects of urban areas were possibly associated with urban greening [45]. However, in the present study, only a few plants were observed in the urban area, and the restorativeness of the nighttime urban environment did not seem to be strongly associated with plants. Han [70] reported that in addition to the scenic beauty, the restorativeness was also related to viewers’ preference, which may help to understand our results. Our interview results showed that nearly half of the participants preferred the urban environment in the nighttime walking (Figure 11), which might be the reason for the similar restorative effects in the urban area and green space. Overall, our findings imply that in addition to natural environments, other artificial environments may also be positive and restorative in a specific population or under certain conditions.

### 4.4. Limitations

With intriguing findings in the present study, there are still some limitations. Firstly, because of the non-normally distributed data, the diurnal variation of measured physiological parameters, we could not explore the interaction between time and sites. Secondly, detailed physiological responses such as stress hormones and brain waves were not investigated due to the limited experimental conditions. Therefore, the mechanisms of benefits in in different walking scenarios were not further discussed. Thirdly, the main reason to walk in green environments is the air purification function of trees [71,72]. However, the experiment was carried out in October, which is just in the rainy season of Chengdu Plain, and the air pollution was at very low levels during the whole experiment (Table 3), so the influences of air pollution were not investigated, which limits us to generalize our findings to less polluted periods or areas. Besides, the impact of air pollution on the nighttime walking and whether a small green space such as a greenway can purify air need to be verified in future studies. Lastly, we ran the physiological and psychological tests immediately after the walking program, therefore only the acute effects were considered, and the long-term effects may remain a future topic.

## 5. Conclusions

This study provided scientific evidence for the physiological and psychological effects of walking in urban areas and green spaces. Our findings suggest that the daytime green environments are advantageous to mental relaxation and can help to lower blood pressure, while the urban environments are negatively associated with walking exercise and may attenuate positive effects of physical activities. However, the urban area may become attractive and show positive effects in nighttime walking, while the psychological influences may be subtle during the nighttime green walking, and nighttime walking in both urban areas and green spaces may provide similar benefits. Taking into account the limitations of the present study, we would recommend the urban citizens start nighttime green walking after work, and nighttime urban walking is also advisable when the air is less polluted.

## Figures and Tables

**Table 1:** Basic information of participants from each group.

Groups	Number	Age	BMI Index
Group1 (DG)	12 (5 males)	56.92 ± 2.29	36.30 ± 0.98
Group2 (NG)	12 (4 males)	51.50 ± 2.61	36.16 ± 1.16
Group3 (DU)	12 (3 males)	56.41 ± 2.79	34.79 ± 1.55
Group3 (NU)	12 (4 males)	53.42 ± 2.60	35.95 ± 1.31

Note: Data are expressed as mean ± stand error (*N* = 12).

**Table 2:** Process and test contents of the experiment.

Time	Daytime Walking (3:30–5:00 PM)		Nighttime Walking (8:30–10:00 PM)	
Day 1	Green Walking		Green Walking	
Day 2	Urban Walking		Urban Walking	
Test	Psychological Item	Measuring Time	Physiological Item	Measuring Time
Pre-test	PANAS, POMS	15 min before walk	SBP, DBP, Pulse rate	Just before walk
Post-test	PANAS, POMS, PRS, ROS	Immediately after walk	SBP, DBP, Pulse rate	15 min after walk

**Table 3:** The environmental parameters in the experiment.

Group	Illumination (lx)	Humidity (%)	Temperature (°C)	Noise (dB)	AQI
DG	3510.67 ± 192.21b	63.27 ± 1.4a	21.73 ± 0.21a	46.33 ± 1.23a	18
NG	8 ± 3.64a	71.07 ± 1.46b	19.02 ± 0.21c	45.33 ± 1.87a	23
DU	7982.5 ± 1642.74c	67.83 ± 1.16b	19.6 ± 0.13b	60.83 ± 2.12c	19
NU	29.5 ± 7.41a	78.25 ± 0.73c	17.5 ± 0.14d	52.17 ± 0.54b	25

Note: The DG, NG, DU, and NU represent daytime green walking, nighttime green walking, daytime urban walking, and nighttime urban walking, respectively. AQI represents Air Quality Index, and AQI < 50 means excellent air quality. Data are expressed as mean ± stand error. Different lowercase letters after the numbers represent statistically significant differences in different trials (*p* < 0.05), analyzed using one-way ANOVA and Duncan’s multiple range test (*N* = 6).

**Table 4:** Internal consistency of the scales in each scenario.

Parameter	Cronbach’s α			
	DG	NG	DU	NU
PANAS	0.76	0.77	0.82	0.73
POMS	0.56	0.86	0.94	0.84
PRS	0.95	0.88	0.96	0.80
ROS	0.62	0.69	0.78	0.93

Note: The DG, NG, DU, and NU represent daytime green walking, nighttime green walking, daytime urban walking, and nighttime urban walking, respectively.

**Table 5:** Proportions of participants with positive responses in psychological parameters after walking in different scenarios.

Psychological Parameter	DG	NG	DU	NU	*p*
PANAS-Positive	9 (75%)	7 (58.3%)	3 (25%)	7 (58.3%)	*p* = 0.084
PANAS-Negative	3 (25%)	1 (8.3%)	1 (8.3%)	1 (8.3%)	*p* = 0.693
POMS-TMD	8 (66.7%)	8 (66.7%)	3 (25%)	11 (91.7%)	*p* = 0.10

Note: The DG, NG, DU, and NU represent daytime green walking, nighttime green walking, daytime urban walking, and nighttime urban walking respectively. Analyzed using the Fisher’s exact test.

**Table 6:** Results of two-way ANOVA for ROS outcomes.

	*df*	F	*p*
Site	1	16.418	<0.001
Time	1	0.701	0.407
Site × Time	1	4.885	0.032

**Table 7:** Proportions of participants with positive responses in measured parameters after walking in different scenarios.

Physiological Parameter	DG	NG	DU	NU	*p*
SBP	11 (91.7%) a	11 (91.7%) a	4 (33.3%) b	9 (75%) ab	*p* = 0.002
DBP	11 (91.7%) a	11 (91.7%) a	5 (41.7%) a	11 (91.7%) a	*p* = 0.005
MAP	11 (91.7%) a	11 (91.7%) a	4 (33.3%) b	10 (83.3%) ab	*p* = 0.004
Pulse rate	6 (50%) a	8 (66.7%) a	7 (58.3%) a	9 (75%) a	*p* = 0.753

Note: The DG, NG, DU, and NU represent daytime green walking, nighttime green walking, daytime urban walking, and nighttime urban walking, respectively. Lowercase letters indicate significant difference (*p* < 0.05). Analyzed using the Fisher’s exact test.

## Data Availability

The data presented in this study are available on request from the corresponding author. The data are not publicly available due to privacy.
